# Mild decrease in heart rate during early phase of targeted temperature management following tachycardia on admission is associated with unfavorable neurological outcomes after severe traumatic brain injury: a post hoc analysis of a multicenter randomized controlled trial

**DOI:** 10.1186/s13054-018-2276-6

**Published:** 2018-12-19

**Authors:** Akihiko Inoue, Toru Hifumi, Yasuhiro Kuroda, Naoki Nishimoto, Kenya Kawakita, Susumu Yamashita, Yasutaka Oda, Kenji Dohi, Hitoshi Kobata, Eiichi Suehiro, Tsuyoshi Maekawa, on behalf of the Brain Hypothermia (B-HYPO) Study Group in Japan

**Affiliations:** 1 Department of Emergency, Disaster and Critical Care Medicine, Kagawa University Hospital, 1750-1 Ikenobe, Miki, Kita, Kagawa 761-0793 Japan; 2 Department of Emergency and Critical Care Medicine, Hyogo Emergency Medical Center, 1-3-1 Wakinohamakaigandori, Chuo-ku, Kobe, Hyogo 651-0073 Japan; 3 Emergency and Critical Care Medicine, St. Luke’s International Hospital, 9-1 Akashi-cho, Chuo-ku, Tokyo, 104-8560 Japan; 4 Clinical Research and Medical Innovation Center, Hokkaido University Hospital, Kita 14, Nishi 5, Kita-ku, Sapporo, Hokkaido 060-8648 Japan; 5 Department of Emergency Medicine, Tokuyama Central Hospital, 1-1 Kouda, Shunan, Yamaguchi, 745-8522 Japan; 6 Advanced Medical Emergency and Critical Care Center, Yamaguchi University School of Medicine, 1-1-1 Minami Kogushi, Ube, Yamaguchi, 755-8505 Japan; 7 Department of Emergency, Disaster and Critical Care Medicine, Showa University, 1-5-8 Hatanodai, Shinagawa-ku, Tokyo, 142-8555 Japan; 8 Osaka Mishima Emergency Critical Care Center, 11-1 Minamiakutagawacho, Takatsuki, Osaka, 569-1124 Japan; 9 Yamaguchi Prefectural University, 3-2-1 Sakurabatake, Yamaguchi City, Yamaguchi 753-8502 Japan

**Keywords:** Traumatic brain injury, Admission heart rate, Heart rate change, Targeted temperature management, Neurological outcomes

## Abstract

**Background:**

The association between isolated admission heart rate (HR) and prognosis has been discussed, but not that between gross HR change and neurological outcome in patients with severe traumatic brain injury (TBI). In the acute phase of severe TBI, HR is influenced by several factors (e.g., pain, sympathetic activation, hypovolemia, fever, body temperature). Therefore, admission HR and gross HR change should be examined in patients with TBI treated with a well-designed protocol, such as was done in the Brain Hypothermia (B-HYPO) Study.

**Methods:**

This was a post hoc analysis of the B-HYPO Study, which was conducted as a prospective, multicenter, randomized controlled trial in patients with severe TBI receiving mild therapeutic hypothermia (MTH; 32.0 °C–34.0 °C) or fever control (35.5 °C–37.0 °C) in Japan. Patients with MTH were examined, and HR change (%HR) in the early MTH phase was calculated as follows: [admission HR – HR at day 1]/admission HR × 100. Patients were divided into six groups, using admission HR (< 80, 80–99, ≤ 100) and median of %HR; i.e., group (Admission HR < 80 and %HR ≥ 18.6); group (Admission HR < 80 and %HR < 18.6); group (Admission HR 80–99 and %HR ≥ 18.6); group (Admission HR 80–99 and %HR < 18.6); group (Admission HR ≥100 and %HR ≥ 18.6); and group (Admission HR ≥100 and %HR < 18.6). The primary outcome was an adjusted predicted probability of unfavorable neurological outcome at 6 months after TBI according to Glasgow Outcome Scale score, which is a measure of functional recovery and defined as severe disability, persistent vegetative state, and death.

**Results:**

Overall, 79 patients with MTH (52.7% of the original trial) were examined; among these, unfavorable neurological outcomes were observed in 53.2%. Among all the groups, group (Admission HR ≥100 and %HR < 18.6) exhibited the highest proportion of unfavorable outcomes, and 82.3% of patients had an adjusted predicted probability of unfavorable outcomes, whereas those in group (Admission HR < 80 and %HR ≥ 18.6) developed only 22.8% (*p* = 0.04).

**Conclusions:**

Mild HR decrease during the early phase of targeted temperature management following tachycardia at admission can be associated with unfavorable neurological outcomes after severe TBI.

**Electronic supplementary material:**

The online version of this article (10.1186/s13054-018-2276-6) contains supplementary material, which is available to authorized users.

## Background

Several studies have reported the association between bradycardia during targeted temperature management (TTM) and good neurological outcome in comatose survivors of out-of-hospital cardiac arrest (OHCA) [1–3]. Therefore, attention has been focused on the association between heart rate (HR)/HR change during TTM and neurological outcome in neurocritical care.

HR has been discussed as an autonomic dysfunction in patients with severe traumatic brain injury (TBI). An association between isolated admission HR and prognosis has been discussed [4, 5], as has the association between exposure to beta-blockers and mortality in patients with TBI, but the studies did not focus on HR change [6, 7]. Recently, HR variability (i.e., tiny HR change) has been reported to be associated with increased mortality after TBI [8]. Therefore, the association between gross HR change during the early phase of TTM and neurological outcomes in patients with severe TBI must be examined.

In the acute phase of severe TBI, HR is influenced by several factors, such as pain [9], sympathetic activation [10], hypovolemia caused by massive bleeding from other injured sites [11], fever [12], and body temperature [13]. Therefore, admission HR and HR change should be examined in patients with severe TBI treated with a well-designed protocol in which sedation, analgesia, target body temperature, blood volume, and treatment of injured organs were well controlled. We describe the association between HR change during the early phase of TTM and unfavorable neurological outcomes in patients with severe TBI using data from the Brain Hypothermia (B-HYPO) Study Group, in which the primary outcome was Glasgow Outcome Scale (GOS) score at 6 months [14].

## Methods

### B-HYPO Study

The B-HYPO Study was conducted as a prospective, multicenter, randomized controlled trial (RCT) between December 2002 and September 2008 in Japan. The protocol was approved by the institutional review board of each participating hospital, and the trial was registered at the University Hospital Medical Information Network site (UMIN-CTR no. C000000231) in Japan and at the National Institutes of Health site (ClinicalTrials.gov identifier NCT00134472) in the United States. In brief, inclusion criteria were age 15 to 69 years for both sexes and a Glasgow Coma Scale (GCS) score of 4 to 8 measured upon arrival at the hospital. Written informed consent was obtained from legally authorized representatives of patients before inclusion. If informed consent could not be obtained within 2 h of admission, the consent policy was waived.

### Patients

**Fig. 1:**
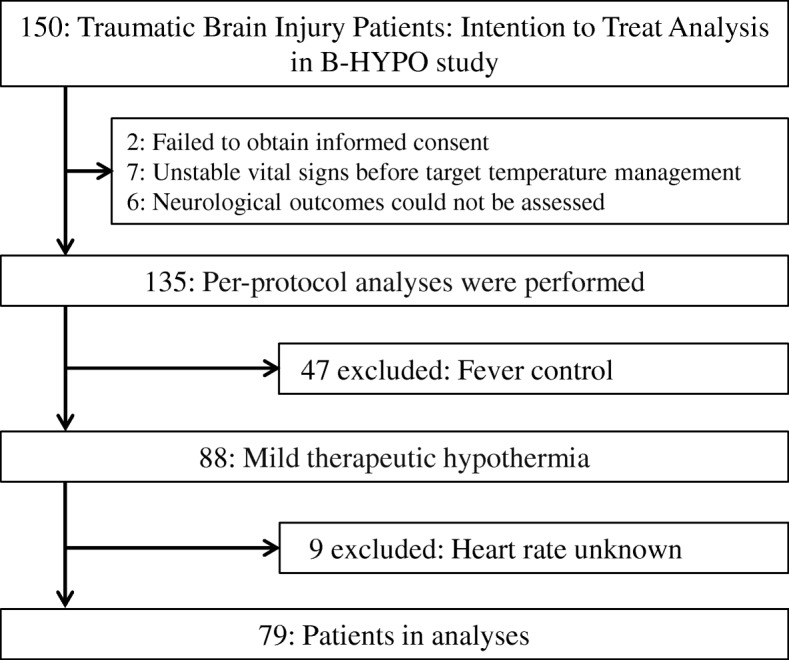
Patient flow

In the original study, 150 patients were assigned randomly (1:2 allocation ratio) to either the fever control (35.5 °C–37.0 °C) or mild therapeutic hypothermia (MTH) group (32.0 °C–34.0 °C), and they were analyzed by intention-to-treat analysis [14]. Per-protocol analysis was performed in 135 patients (Fig. 1) (fever control, 47 patients; MTH, 88 patients) [15]. In the present post hoc study, we described data of these patients with MTH (*n* = 88) on the basis of HR change between admission and day 1.

### Targeted temperature management

Treatments were performed as described in our original study [14]. In brief, cooling was initiated within 2 h of the onset of TBI. Cooling blankets, rapid cold fluid infusion (up to 1000 ml of saline, human plasma products, or dextrose-free plasma expanders), and/or cold gastric lavage were used during the induction phase in both groups. The goal in each group was to achieve the targeted temperature within 6 h of the onset of TBI and to maintain this temperature for at least 72 h, predominantly using surface cooling blankets. After 72 h, the temperature was maintained at < 38 °C until 7 days after the onset of TBI.

### Sedation and hemodynamic management

The sedation protocol specified either midazolam (0.2–0.4 mg/kg/h) and nonnarcotic analgesics or neuroleptic analgesia (25 μg/kg/h droperidol and 1 μg/kg/h fentanyl). Sedatives and analgesics were usually tapered once the patients had been rewarmed to 36 °C. Neuromuscular blockade, including vecuronium (0.05 mg/kg/h) or pancuronium (0.05 mg/kg/h), was used during induction and maintenance phases as necessary. Shivering, if it developed, was evaluated and managed according to the criteria of each facility.

Hemodynamic status was monitored and maintained strictly, using an arterial catheter, pulmonary arterial catheter, and intracranial pressure (ICP) monitoring probe to monitor hemodynamic status and ICP at the following levels: mean arterial pressure > 80 mmHg, cardiac index > 2.5 L/min/m^2^, systemic vascular resistance index 800 to 1200 dyn/s/cm^5^, ICP < 20 mmHg, and cerebral perfusion pressure > 60 mmHg.

### Data collection and study outcomes

Data on the following parameters were collected: age, sex, HR, blood pressure, GCS score, unreactive pupil or pupils on admission, Traumatic Coma Data Bank computed tomography classification [16], surgical intervention for TBI during admission, ICP, Injury Severity Score (ISS), Abbreviated Injury Scale score for the head, blood glucose, TTM (MTH or fever control), and unfavorable neurological outcomes at 6 months following TBI. MTH was achieved at a median of 8.1 h (IQR, 5.3–11.8 h) [14]. HR was measured at admission (Admission HR) and on day 1 (median time, 23.4 h after admission).

### Primary exposure

HR change (%HR) between Admission HR and HR at early stage of TTM (23.4 h, 17.4–28.7 h, in the B-HYPO Study) was calculated as (%HR = [admission HR – HR at day 1]/admission HR × 100). Because the HR change (admission HR – HR at day 1) is considerably influenced by admission HR, we used %HR instead of ΔHR to measure HR change. A positive value shows a decrease in HR, whereas a negative value shows an increase in HR from admission to day 1. That is, a larger %HR corresponds with a moderate decrease, whereas a smaller %HR corresponds with a mild decrease in HR from admission to day 1.

A previous study examining the association between admission HR and mortality in patients with moderate to severe TBI reported a smooth U-shaped relationship between admission HR and mortality, with the lowest mortality in patients with HR 80 to 99. Therefore, we used three cutoff values for admission HR (< 80, 80–99, ≤ 100) [4]. With regard to %HR, we used the median of %HR 18.6 because there were no previous studies examining the HR change in patients with TBI. Thus, to describe the association between admission HR or %HR and unfavorable neurological outcomes, study patients were divided into six groups using the admission HR (< 80, 80–99, ≤ 100) and median %HR (median, 18.6; IQR, − 8.6 to 32.5): group (Admission HR < 80 and %HR ≥ 18.6), group (Admission HR < 80 and %HR < 18.6), group (Admission HR 80–99 and %HR ≥ 18.6), group (Admission HR 80–99 and %HR < 18.6), group (Admission HR ≥ 100 and %HR ≥ 18.6), and group (Admission HR ≥ 100 and %HR < 18.6).

### Study endpoints

Primary outcome was an adjusted predicted probability of unfavorable neurological outcome at 6 months after TBI, where an unfavorable outcome was defined as severe disability, persistent vegetative state, and death, according to the GOS score, which is a measure of functional recovery.

### Statistical analyses

To compare baseline characteristics, study participants were divided into six groups using the primary exposure. Next, because of the small number of patients included in the present study, instead of performing multiple analyses to examine whether HR change (i.e., in the six groups) could be an independent predictor of unfavorable outcome, we used the multiple logistic regression models adjusting for age [17–19], sex [20], GCS score [17, 21, 22], unreactive pupil on admission [23], surgical intervention for TBI during admission [24], ICP [25] at day 1, and ISS [17, 22] to obtain adjusted predicted probabilities of unfavorable outcome in the six different groups using the admission HR and median %HR; therefore, six HR change groups were not included in the analyses as adjusting factors.

Continuous variables were analyzed using the Mann-Whitney *U* test or Kruskal-Wallis test, and categorical comparisons were performed using the χ^2^ or Fisher’s exact test, when appropriate. Statistical analysis was performed using JMP version 12 statistical software (SAS Institute, Inc., Cary, NC, USA). Results are presented as *n* (%) or median (IQR). *P* < 0.05 was considered statistically significant.

## Results

**Fig. 2:**
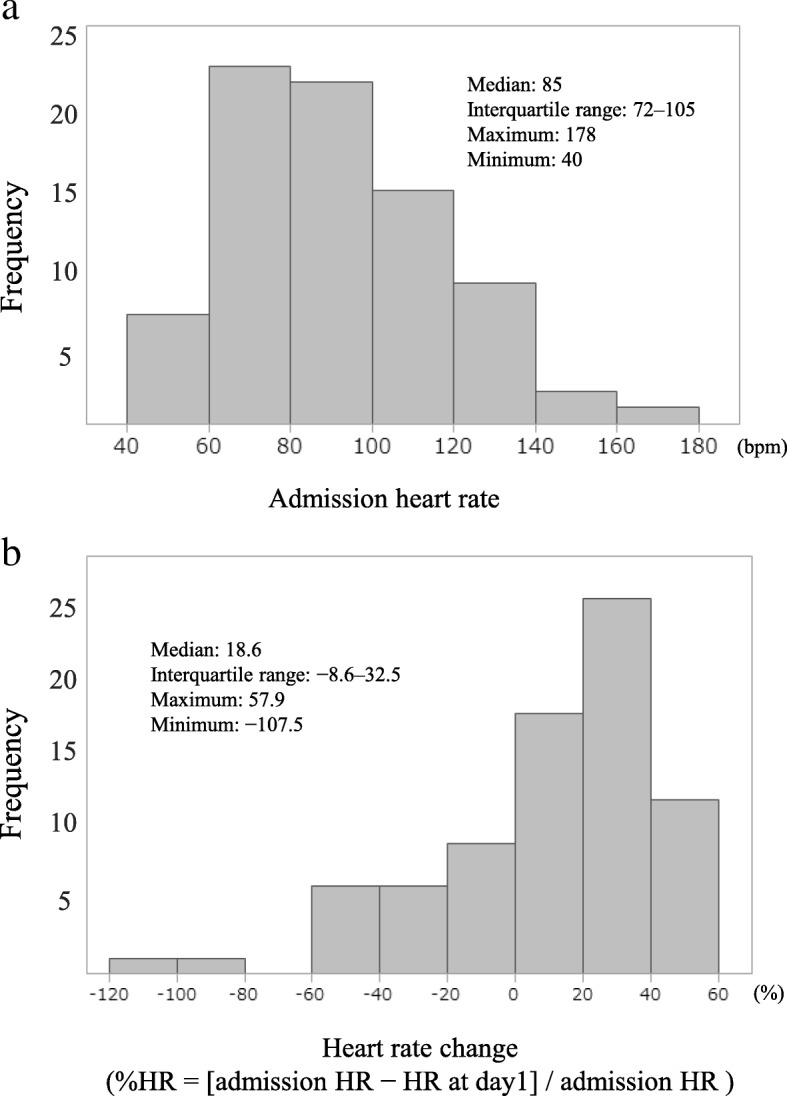
**a** Distribution of admission heart rate. **b** Distribution of heart rate change. *Bpm* Beats/min, *HR* Heart rate

**Fig. 3:**
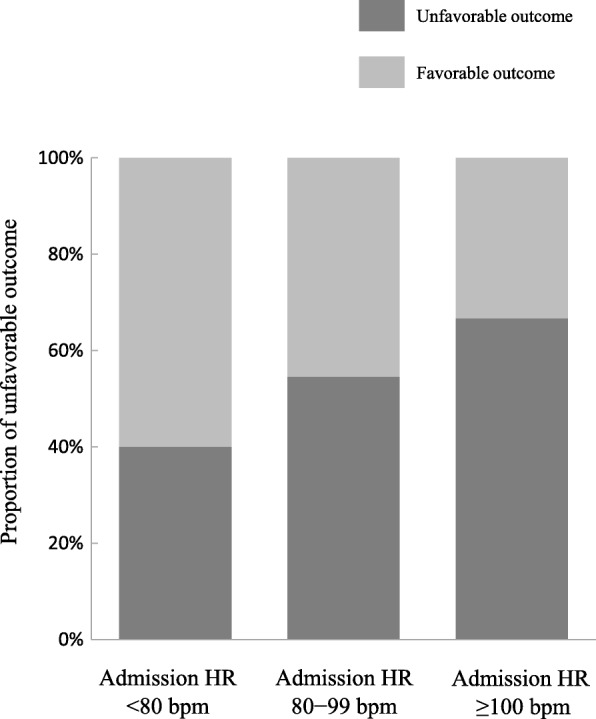
Associations between unfavorable outcome and admission heart rate. The proportions of unfavorable outcome were 40.0%, 54.5%, and 66.7% in patients with admission HR < 80 bpm, admission HR 80–99 bpm, and admission HR ≥ 100 bpm, respectively. An unfavorable outcome was defined as severe disability, persistent vegetative state, and death, whereas a favorable outcome was defined as moderate disability or good recovery, according to the Glasgow Outcome Scale scores. *HR* Heart rate, *bpm* Beats/min

A total of nine patients were excluded owing to having an unavailable admission HR or day 1 HR. The remaining 79 patients (median age 40.0 years, 70.1% male) were analyzed (Fig. 1). Unfavorable neurological outcomes and survival rates at 6 months were 53.2% and 65.8%, respectively, and 28.8% (15/52) of actual survivors had an unfavorable outcome. The median GCS score was 6 (4–7), and median ISS was 25 (17–34) (Table 1). The median Admission HR was 85 beats/min [bpm] (72–105), and median %HR was 18.6 (− 8.6 to 32.5). The distribution of these data is shown in Fig. 2a and b. The proportions for unfavorable outcome were 40.0%, 54.5%, and 66.7% in patients with Admission HR < 80 bpm, Admission HR 80–99 bpm, and Admission HR ≥ 100 bpm, respectively (Fig. 3). The baseline characteristics were significant differences in age and ISS (Table 1). Other detailed data of baseline characteristics were divided into six groups using primary exposure and are shown in Additional file 1: Table S1. There were significant differences in blood glucose at day 1, stress index at day 1, and pulmonary arterial wedge pressure at day 1. Comparison of patient characteristics between the unfavorable and favorable outcome groups showed significant differences in age (Additional file 1: Table S2). Fever control patients (*n* = 47) were excluded from the present study. A comparison of patient characteristics between the MTH and fever control groups is shown in Additional file 1: Table S3.

**Table 1:** Patient characteristics

Variables	Total (*n* = 79)	Admission HR < 80		Admission HR 80–99		Admission HR ≥ 100		*P* value
		%HR ≥ 18.6	%HR < 18.6	%HR ≥ 18.6	%HR < 18.6	%HR ≥ 18.6	%HR < 18.6	
		(*n* = 7)	(*n* = 23)	(*n* = 11)	(*n* = 11)	(*n* = 22)	(*n* = 5)	
Age (years)	40 (21–57)	27 (17–57)	46 (22–62)	55 (51–68)	21 (17–45)	28 (21–55)	54 (28–63)	0.02
Male sex (%)	54 (70.1)	6 (100)	16 (69.6)	7 (63.6)	6 (60.0)	17 (77.3)	2 (40.0)	0.31
Vital signs								
SBP on admission (mmHg)	140 (110–170)	154 (130–160)	160 (120–186)	148 (130–187)	112 (100–146)	131 (109–166)	140 (109–151)	0.06
SBP at day 1 (mmHg)	124 (108–145)	132 (116–143)	124 (104–145)	138 (120–147)	126 (110–146)	119 (105–150)	120 (103–147)	0.87
GCS score	6 (4–7)	6 (5–7)	6 (5–7)	6 (4–7)	5 (4–7)	6 (4–6.3)	5 (4–7)	0.93
4–5	34 (43.0)	3 (42.9)	9 (39.1)	5 (45.5)	6 (54.6)	8 (36.4)	3 (60.0)	0.88
6–8	45 (57.0)	4 (57.1)	14 (60.9)	6 (54.6)	5 (45.5)	14 (63.6)	2 (40.0)	
Unreactive pupil or pupils on admission (%)	38 (50.0)	6 (85.7)	12 (54.6)	7 (70.0)	4 (36.4)	6 (28.6)	3 (60.0)	0.07
TCDB CT classification (%)								0.681
Diffuse injury grade I	1 (1.8)	0	0	0	0	0	0	
Diffuse injury grade II	21 (26.6)	2 (9.5)	5 (23.8)	3 (14.3)	1 (4.8)	9 (42.9)	1 (4.8)	
Diffuse injury grade III	11 (13.9)	0	2 (18.2)	3 (27.3)	2 (18.2)	3 (27.3)	1 (9.1)	
Diffuse injury grade IV	2 (2.5)	0	2 (100)	0	0	0	0	
Evacuated mass	39 (49.4)	4 (10.3)	14 (35.9)	5 (12.8)	7 (18.0)	7 (18.0)	2 (5.1)	
Nonevacuated mass	5 (6.3)	1 (20.0)	0	0	1 (20.0)	2 (40.0)	1 (20.0)	
Surgical operation for TBI (%)	42 (53.2)	4 (57.1)	17 (73.9)	5 (45.5)	7 (63.6)	8 (36.4)	1 (20.0)	0.09
Hemodynamic parameter								
Initial ICP (mmHg)	14 (7–34)	15 (4–16)	24 (7–40)	13 (7–25)	34 (11–51)	11 (7–19)	8 (4–77)	0.40
ICP at day 1 (mmHg)	14 (10–23)	12 (11–19)	15 (11–33)	13 (10–21)	31 (11–66)	13 (7–18)	18 (8–96)	0.19
ISS	25 (17–34)	24 (16–25)	25 (16–34)	24 (19–36)	34 (26–38)	29 (18–35)	25 (15–33)	0.04
AIS for head								0.24
3–4	40 (54.1)	4 (57.1)	14 (63.6)	7 (70.0)	2 (20.0)	10 (50.0)	3 (60.0)	
5	34 (46.0)	3 (42.9)	8 (36.4)	3 (30.0)	8 (80.0)	10 (50.0)	2 (40.0)	
Unfavorable outcome^a^ (%)	42 (53.2)	3 (42.9)	9 (39.1)	5 (45.5)	7 (63.6)	13 (59.1)	5 (100)	0.18
Survive (%)	52 (65.8)	6 (85.7)	15 (65.2)	8 (72.7)	7 (63.6)	15 (68.2)	1 (20.0)	0.29

*Abbreviations: Admission HR* Admission heart rate, *%HR* Heart rate change [admission HR – HR at day 1]/admission HR × 100, *SBP* Systolic blood pressure, *GCS* Glasgow Coma Scale *TCDB* Traumatic Coma Data Bank *CT* Computed tomography *TBI* Traumatic brain injury *ICP* Intracranial pressure *ISS* Injury Severity Score *AIS* Abbreviated Injury Scale *TTM* Targeted temperature management

Values are presented as medians (IQR) or number of patients (percent)

^a^ Unfavorable outcome was defined as severe disability, persistent vegetative state, and death according to Glasgow Outcome Scale scores

### Association between %HR and unfavorable neurological outcomes

**Fig. 4:**
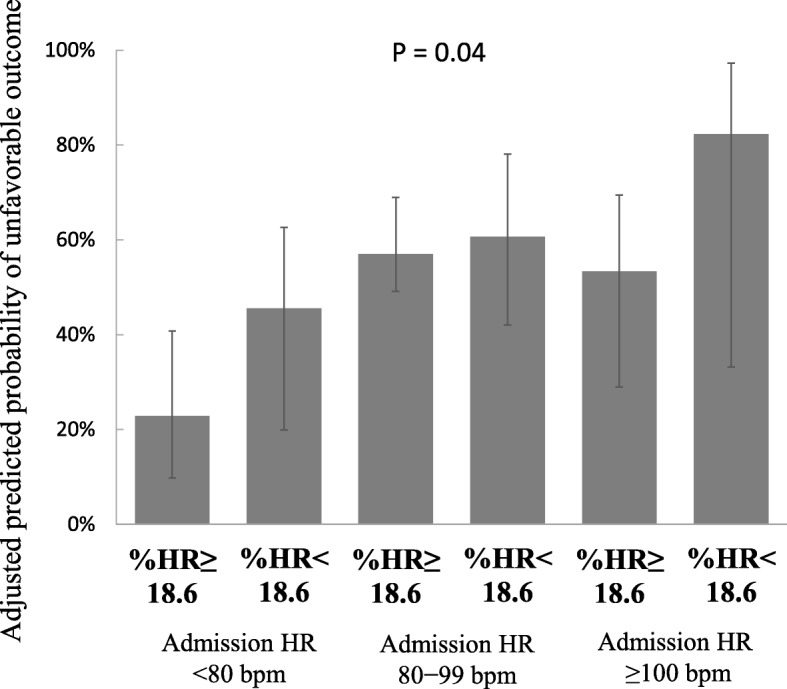
The adjusted predicted probability of unfavorable outcome for %HR groups. The median adjusted predicted probabilities of unfavorable outcome were 22.8%, 45.6%, 57.0%, 60.7%, 53.4%, and 82.3% in group (Admission HR < 80 and %HR ≥18.6), group (Admission HR < 80 and %HR < 18.6), group (Admission HR 80–99 and %HR ≥18.6), group (Admission HR 80–99 and %HR < 18.6), group (Admission HR ≥100 and %HR ≥18.6), and group (Admission HR ≥100 and %HR < 18.6), respectively. *HR* Heart rate, *%HR* Heart rate change ([admission HR – HR at day 1]/admission HR × 100), *bpm* Beats/min. Error bars indicate the IQR

Regarding the primary endpoint, group (Admission HR ≥ 100 and %HR < 18.6) had the highest proportion (100%) of unfavorable outcomes among the six groups (Table 1). The adjusted predicted probabilities of unfavorable outcome were 22.8%, 45.6%, 57.0%, 60.7%, 53.4%, and 82.3% in group (Admission HR < 80 and %HR ≥ 18.6), group (Admission HR < 80 and %HR < 18.6), group (Admission HR 80–99 and %HR ≥ 18.6), group (Admission HR 80–99 and %HR < 18.6), group (Admission HR ≥ 100 and %HR ≥ 18.6), and group (Admission HR ≥ 100 and %HR < 18.6), respectively (Fig. 4).

## Discussion

In the present post hoc study, unfavorable neurological outcomes occurred in 53.2% (42 of 79) patients with severe TBI. Group (Admission HR ≥ 100 and %HR < 18.6) had the highest proportion of unfavorable outcomes, and 82.3% of those patients had an adjusted predicted probability of unfavorable outcome, whereas group (Admission HR < 80 and %HR ≥ 18.6) developed only 22.8%. In the present study, we limited the patients to only those in the MTH group because the difference in targeted temperature may cause strong heterogeneity in HR change.

Two previous studies demonstrated the association between isolated admission HR and mortality [4, 5]. A smooth U-shaped relationship was observed between admission HR and mortality, with the lowest mortality in patients with HR 80 to 89 [4, 5]. In the present study, patients with an Admission HR ≥ 100 bpm followed by %HR < 18.6 during initiation of TTM demonstrated an 82.3% adjusted predicted probability of an unfavorable outcome, whereas patients with %HR ≥ 18.6 in those patients had an approximately 50% of predicted probability of an unfavorable outcome. These facts suggested that tachycardia at admission followed by mild decrease in HR during the early phase of TTM could be another candidate for predicting unfavorable neurological outcomes.

During MTH, it has been well discussed that the suppression of HR was caused by suppression of spontaneous depolarization of cardiac pacemaker cells, prolongation of the duration of action potentials, slowing of myocardial impulse conduction [13], indirect suppression of sympathetic activity [26–28], and activation of parasympathetic activity [28]. Sympathetic activation also is an important factor in %HR during TTM. We suspected that Admission HR ≥ 100 bpm reflected sympathetic activation, high plasma catecholamine level, and severity of primary damage in patients with TBI. The reduction of %HR reflected the reduction of plasma catecholamine levels in patients whose Admission HR had increased to ≥ 100 bpm [29]. Therefore, we considered that patients with tachycardia on admission followed by mild decrease in HR during early-phase TTM had a high incidence of unfavorable outcomes.

Many RCTs have been conducted to investigate the effectiveness of MTH for TBI, but they could not demonstrate more favorable outcomes than those obtained by normothermia (at 37 °C) [14, 30–32]. However, the latest guidelines from an expert panel suggest considering TTM at 35 °C–37 °C to improve survival with good neurological outcome in patients with severe TBI, and also considering TTM at 34 °C–35 °C to lower ICP in patients with TBI with refractory intracranial hypertension despite medical treatments [33]. Thus, TTM (mild hypothermia and fever control) should be considered in patients with severe TBI. In such situations, withdrawal of intensive care always should be considered after initial TTM, because recent guidelines on OHCA primarily address the termination of resuscitative efforts during performance of TTM [34, 35]. Appropriate determination of factors predicting neurological outcomes also may contribute to reduce healthcare-associated costs. According to our results, all patients in group (Admission HR ≥ 100 bpm and %HR < 18.6) had unfavorable outcomes.

There are several limitations to our study. First, the original study was terminated before the full sample size was reached. Additionally, the sample size was reduced further from 150 to 79 patients because HR could not be obtained in 9 to 88 patients. These factors may have biased the outcomes of our study. Second, confounders of HR response, such as the use of preinjury beta-blockers [1, 36, 37], vasopressor support, amount of bleeding and fluids, and urine volume, were not examined, owing to unavailability of the dataset. However, hemodynamic status was monitored and maintained based strictly on the study protocol. Third, the number of patients included in the present study was small. Furthermore, we divided included patients into six groups using the admission HR (< 80, 80–99, ≤ 100) and median %HR (median 18.6), which might have caused complexity. Finally, selection bias may have been present.

## Conclusions

Mild decrease in HR during initiation of TTM following an initially increased HR can be associated with unfavorable neurological outcomes after severe TBI.

## Additional file

Additional file 1: **Table S1.** Patient characteristics. **Table S2.** Comparison of patient characteristics between unfavorable and favorable outcomes. **Table S3.** Comparison of patient characteristics between the mild therapeutic hypothermia and fever control groups. (DOCX 44 kb)

## Abbreviations

AIS: Abbreviated Injury Scale
B-HYPO Study: Brain Hypothermia Study
CT: Computed tomography
GCS: Glasgow Coma Scale
GOS: Glasgow Outcome Scale
HR: Heart rate
ICP: Intracranial pressure
ISS: Injury Severity Score
MTH: Mild therapeutic hypothermia
OHCA: Out-of-hospital cardiac arrest
RCT: Randomized controlled trial
TBI: Traumatic brain injury
TCDB: Traumatic Coma Data Bank
TTM: Targeted temperature management
