# Supplementary material for: Mild decrease in heart rate during early phase of targeted temperature management following tachycardia on admission is associated with unfavorable neurological outcomes after severe traumatic brain injury: a post hoc analysis of a multicenter randomized controlled trial
Source: Crit Care. 2018 Dec 19;22:352. doi: 10.1186/s13054-018-2276-6 (PMC6300018; doi:10.1186/s13054-018-2276-6)
Supplement: Supplementary file 1 — Table S1. Patient characteristics. Table S2. Comparison of patient characteristics between unfavorable and favorable outcomes. Table S3. Comparison of patient characteristics between the mild therapeutic hypothermia and fever control groups. (DOCX 44 kb) [file 13054_2018_2276_MOESM1_ESM.docx]

**Table S1.** **Patient characteristics**

|  | **Total**  (*n* = 79) | **Admission HR < 80** | | **Admission HR 80–99** | | **Admission HR ≥ 100** | | ***P* value** |
| --- | --- | --- | --- | --- | --- | --- | --- | --- |
| Variables |  | **%HR ≥ 18.6** | **%HR < 18.6** | **%HR ≥ 18.6** | **%HR < 18.6** | **%HR ≥ 18.6** | **%HR < 18.6** |  |
|  |  | **Group 1**  (*n* = 7) | **Group 2**  (*n* = 23) | **Group 3**  (*n* = 11) | **Group 4**  (*n* = 11) | **Group 5**  (*n* = 22) | **Group 6**  (*n* = 5) |  |
| Drugs during TTM |  |  |  |  |  |  |  |  |
| Midazolam (%) | 70 (90.9) | 6 (85.7) | 22 (95.7) | 11 (100) | 10 (90.9) | 18 (90.0) | 3 (60.0) | 0.17 |
| Midazolam dose (mg/h) | 10.0 (5-12.1) | 15.0 (8.8-19.6) | 7.0 (5-12) | 10.0 (5-15) | 10.0 (4.5-10) | 12.0 (7.5-13.3) | 12.0 (2-15) | 0.24 |
| Droperidol (%) | 19 (27.1) | 1 (16.7) | 6 (33.3) | 2 (20.0) | 3 (27.3) | 7 (35.0) | 0 | 0.63 |
| Droperidol dose (mg/h) | 2.5 (1.5-3.6) | 2.0 (2-2) | 3.3 (1.9-10.2) | 5.3 (2.5-8) | 1.8 (1.5-3.4) | 2 (1.3-3.8) | - | NA |
| Fentanyl (%) | 43 (57.3) | 5 (71.4) | 14 (70.0) | 5 (45.5) | 6 (54.6) | 12 (57.1) | 1 (20.0) | 0.37 |
| Fentanyl dose (mg/h) | 0.06 (0.05-0.2) | 0.09 (0.06-1.7) | 0.05 (0.05-0.13) | 0.06 (0.01-0.77) | 0.07 (0.04-0.2) | 0.06 (0.06-0.5) | - | 0.53 |
| Vecuronium (%) | 77 (98.7) | 7 (100) | 23 (100) | 11 (100) | 11 (100) | 20 (95.2) | 5 (100) | 0.74 |
| Laboratory data |  |  |  |  |  |  |  |  |
| Hemoglobin at day0^a^ (g/dL) | 12.5 (10.6-14) | 13.9 (13.0-14.7) | 12.3 (11.3-13.6) | 10.5 (9.8-11.9) | 13.3 (10.3-14.2) | 13.2 (10.9-14.0) | 10.5 (9.7-13.4) | 0.10 |
| Hemoglobin at day1^b^ (g/dL) | 10.1 (9.3-12) | 12.0 (10.4-12.4) | 10.0 (9.3-12.0) | 10.7 (9.3-12.2) | 9.9 (9.3-11.6) | 10.1 (8.7-11.0) | 9.8 (8.1-14.2) | 0.44 |
| Hematocrit at day0 (%) | 37.7 (33-41.3) | 41.6 (38.9-43.6) | 37.6 (33.8-40.7) | 32.3 (29.8-37.1) | 40.7 (31.9-42.5) | 39.0 (34.4-41.6) | 35.7 (28.7-39.3) | 0.11 |
| Hematocrit at day1 (%) | 30.3 (27.1-34.1) | 34.7 (29.6-36.9) | 31.7 (27.0-34.1) | 30.3 (27.5-33.4) | 29.0 (27.2-31.8) | 30.0 (25.1-35.7) | 29.4 (24.8-41.9) | 0.54 |
| Potassium at day0 (mEq/L) | 3.3 (3.0-3.7) | 3.6 (3.5-3.9) | 3.2 (3.1-3.8) | 3.5 (2.9-4.0) | 3.3 (3.0-3.5) | 3.4 (3.0-3.7) | 3.0 (2.6-3.6) | 0.41 |
| Potassium at day1 (mEq/L) | 3.5 (3.2-3.7) | 3.4 (3.4-3.6) | 3.5 (3.3-3.9) | 3.4 (3.0-3.7) | 3.2 (3.0-3.7) | 3.5 (3.4-4.0) | 3.2 (2.9-3.6) | 0.08 |
| Blood glucose at day0 (mg/dL) | 174 (148-208) | 138 (117-188) | 171 (155-192) | 173 (129-213) | 172 (139-190) | 176 (155-225) | 240 (160-288) | 0.20 |
| Blood glucose at day1 (mg/dL) | 161 (123-211) | 119 (96-166) | 179 (134-206) | 155 (122-173) | 185 (133-226) | 143 (116-187) | 251 (250-291) | 0.03 |
| Stress Index at day0 | 52 (40-64.2) | 38.7 (29.4-53.5) | 53.4 (43.3-56.8) | 54.1 (34.9-62.8) | 49.3 (43.5-54.5) | 48.5 (41.5-78.4) | 77.4 (51.3-107.1) | 0.18 |
| Stress Index at day1 | 45.4 (34.7-57.8) | 34.6 (28.2-46.1) | 48.4 (37.4-59.5) | 43.0 (34.9-60.7) | 53.8 (41.6-57.8) | 40.9 (31.3-50.5) | 83.7 (64.1-90.9) | 0.01 |
| Hemodynamic parameter |  |  |  |  |  |  |  |  |
| CVP at day0 (mmHg) | 4 (3-6) | 3.5 (3.0-4.0) | 6.0 (2.0-8.5) | 6.0 (4.0-7.8) | 5.0 (1.5-8.0) | 4.0 (2.0-5.0) | 5.0 (4.0-6.0) | 0.63 |
| CVP at day1 (mmHg) | 7 (4-8.5) | 8.0 (5.0-10.0) | 6.0 (4.0-9.0) | 8.0 (3.0-12.0) | 6.0 (4.0-7.0) | 6.0 (3.5-8.0) | 4.0 (0-7.8) | 0.57 |
| PAWP at day0 (mmHg) | 11 (7-12) | 9.5 (7-12) | 8.0 (6.5-13) | 9 (7-11.8) | 12 (11-14) | 12 (5-13) | 10 (10-11.5) | 0.88 |
| PAWP at day1 (mmHg) | 9 (7-13) | 12 (10-14) | 8 (7-17.5) | 11.5 (7.5-18.3) | 9 (6-11) | 7 (3-8) | 13.5 (13-18.5) | 0.02 |
| Body temperature (°C) at day 0 | 35.1 (34.1-36.0) | 34.5 (33.0-36.0) | 35.2 (34.6-35.8) | 35.9 (34.3-36.3) | 36.0 (30.2-36.2) | 35.0 (34.0-35.9) | 33.9 (33.9-33.9) | 0.80 |
| Body temperature (°C) at day 1 | 33.2 (32.7-33.6) | 32.7 (32.3-33.4) | 33.5 (32.6-33.8) | 32.9 (32.8-34.0) | 33.3 (32.7-34.1) | 33.2 (32.6-34.9) | 33.2 (33.2-33.2) | 0.88 |

Admission HR, admission heart rate; %HR, heart rate change [admission HR – HR at day 1] / admission HR × 100; TTM, target temperature management; CVP, central venous pressure; PAWP, pulmonary artery wedge pressure; NA, not available.

Stress Index: Blood glucose / potassium.

^a^Day 0: median time was 5.0 h after admission.

^b^Day 1: median time was 23.4 h after admission.

**Table S2. Comparison of patient characteristics between the unfavorable outcome^a^ and favorable outcome^b^**

| Variables | **Unfavorable outcome^a^**  (*n* = 42) | **Favorable outcome^b^**  (*n* = 37) | ***P* value** |
| --- | --- | --- | --- |
| Age (years) | 51.5 (25.8-62.0) | 24.0 (19.0-52.5) | < 0.01 |
| Male (%) | 28 (70.0) | 26 (70.3) | 0.98 |
| Vital signs |  |  |  |
| Admission HR (bpm) | 94(75-106) | 80 (70-103) | 0.22 |
| HR at day 1 (bpm) | 74 (65-91) | 75 (60-88) | 0.71 |
| %HR | 18.8 (-9.0-30.4) | 18.6 (-8.7-34.3) | 0.91 |
| SBP on admission (mmHg) | 145 (120-170) | 130 (110-169) | 0.30 |
| SBP at day1 (mmHg) | 121 (106-146) | 126 (115-144) | 0.67 |
| GCS score | 5.5 (4.0-6.3) | 6.0 (5.0-7.0) | 0.09 |
| Unreactive pupil or pupils on admission (%) | 19 (46.3) | 19 (54.3) | 0.79 |
| TCDB CT classification (%) |  |  | 0.55 |
| Diffuse injury grade I | 0 | 1 (2.7) |  |
| Diffuse injury grade II | 9 (21.4) | 12 (32.4) |  |
| Diffuse injury grade III | 7 (16.7) | 4 (10.8) |  |
| Diffuse injury grade IV | 1 (2.4) | 1 (2.7) |  |
| Evacuated mass | 21 (50.0) | 18 (48.7) |  |
| Non-evacuated mass | 4 (9.5) | 1 (2.7) |  |
| Surgical operation for TBI | 22 (52.4) | 20 (54.1) | 0.88 |
| Hemodynamic parameter |  |  |  |
| Initial ICP (mmHg) | 17 (7-39) | 14 (4-28) | 0.34 |
| ICP at day1 (mmHg) | 18 (10-52) | 13 (10-18) | 0.06 |
| ISS | 26 (20-34) | 25 (17-34) | 0.20 |
| AIS for head | 5 (4-5) | 4 (4-5) | 0.09 |
| Drugs during TTM |  |  |  |
| Midazolam (%) | 38 (90.5) | 32 (91.4) | 0.88 |
| Midazolam dose (mg/h) | 10 (5-12) | 10 (6-14) | 0.37 |
| Droperidol (%) | 8 (21.6) | 11 (33.3) | 0.27 |
| Droperidol dose (mg/h) | 2.3 (1.4-3.7) | 2.5 (1.7-5.6) | 0.70 |
| Fentanyl (%) | 23 (57.5) | 20 (57.1) | 0.98 |
| Fentanyl dose (mg/h) | 0.06 (0.05-0.09) | 0.09 (0.05-0.50) | 0.12 |
| Vecuronium (%) | 41 (97.6) | 36 (100) | 0.35 |

Values are presented as medians (interquartile ranges, IQR), or number of patients (percentile, %).

Admission HR, admission heart rate; Bpm, beats per minute; %HR, heart rate change [admission HR – HR at day 1] / admission HR × 100; SBP, systolic blood pressure; GCS, Glasgow Coma Scale; TCDB, Traumatic Coma Data Bank; CT, computed tomography; TBI, traumatic brain injury; ICP, intracranial pressure; ISS, Injury Severity Score; AIS, Abbreviated Injury Score; TTM, target temperature management.

^a^Unfavorable outcome was defined as severe disability, persistent vegetative state and death according to the Glasgow Outcome Scale scores.

^b^Favorable outcome was defined as s moderate disability and good recovery according to the Glasgow Outcome Scale scores.

**Table S3. Comparison** **of patient characteristics between the mild therapeutic hypothermia and fever control groups.**

| Variables | **Mild therapeutic hypothermia**  (*n* = 79) | **Fever control^a^**  (*n* = 40) | ***P* value** |
| --- | --- | --- | --- |
| Age (years) | 40 (21–57) | 40.5 (22–56.8) | 0.97 |
| Male (%) | 54 (70.1) | 27 (71.1) | 0.92 |
| Vital signs |  |  |  |
| Heart rate on admission | 85 (72–105) | 86 (70–106) | 0.70 |
| Heart rate at day 1 | 75 (60–90) | 81 (65–101) | 0.10 |
| %HR | 18.6 (−8.6–32.5) | 5.9 (−17.3–21.8) | 0.06 |
| SBP on admission (mmHg) | 140 (110–170) | 150 (115–184) | 0.32 |
| SBP at day1 (mmHg) | 124 (108–145) | 130 (114–144) | 0.40 |
| GCS score | 6 (4–7) | 6 (5–7) | 0.59 |
| Unreactive pupil or pupils on admission (%) | 38 (50.0) | 20 (50.0) | 1.00 |
| TCDB CT classification (%) |  |  | 0.50 |
| Diffuse injury grade I | 1 (1.3) | 1 (2.6) |  |
| Diffuse injury grade II | 21 (26.6) | 13 (33.3) |  |
| Diffuse injury grade III | 11 (13.9) | 7 (18.0) |  |
| Diffuse injury grade IV | 2 (2.5) | 2 (5.1) |  |
| Evacuated mass | 39 (49.4) | 16 (41.0) |  |
| Non-evacuated mass | 5 (6.3) | 0 |  |
| Surgical operation for TBI | 42 (53.2) | 21 (52.5) | 0.95 |
| Hemodynamic parameter |  |  |  |
| Initial ICP (mmHg) | 14 (7–34) | 18 (12–44) | 0.13 |
| ICP at day1 (mmHg) | 14 (10–23) | 19 (11–27) | 0.20 |
| ISS | 25 (17–34) | 25 (17–29) | 0.11 |
| AIS for head | 4 (4–5) | 4 (4–5) | 0.28 |
| Drugs during TTM |  |  |  |
| Midazolam (%) | 70 (90.9) | 39 (97.5) | 0.18 |
| Midazolam dose (mg/h) | 10.0 (5.0–12.1) | 10.0 (8.6–16.5) | 0.16 |
| Droperidol (%) | 19 (27.1) | 7 (20.0) | 0.42 |
| Droperidol dose (mg/h) | 2.5 (1.5–3.6) | 3.5 (1.7–13.5) | 0.53 |
| Fentanyl (%) | 43 (57.3) | 21 (55.3) | 0.83 |
| Fentanyl dose (mg/h) | 0.06 (0.05–0.2) | 0.07 (0.05–0.1) | 0.99 |
| Vecuronium (%) | 77 (98.7) | 36 (92.3) | 0.07 |
| Unfavorable outcome^a^ (%) | 42 (53.2) | 19 (47.5) | 0.56 |
| Survive (%) | 52 (65.8) | 30 (75.0) | 0.31 |

Values are presented as medians (interquartile ranges, IQR), or number of patients (percentile, %).

%HR, heart rate change [admission HR – HR at day 1] / admission HR × 100; SBP, systolic blood pressure; GCS, Glasgow Coma Scale; TCDB, Traumatic Coma Data Bank; CT, computed tomography; TBI, traumatic brain injury; ICP, intracranial pressure; ISS, Injury Severity Score; AIS, Abbreviated Injury Score; TTM, target temperature management.

^a^Patients with unknown heart rate and outcomes were excluded.

^b^Unfavorable outcome was defined as severe disability, persistent vegetative state and death according to the Glasgow Outcome Scale scores.
